# Tacrolimus Blood Level Fluctuation Predisposes to Coexisting BK Virus Nephropathy and Acute Allograft Rejection

**DOI:** 10.1038/s41598-017-02140-1

**Published:** 2017-05-16

**Authors:** Chia-Lin Shen, An-Hang Yang, Tse-Jen Lien, Der-Cherng Tarng, Chih-Yu Yang

**Affiliations:** 1 Division of Nephrology, Department of Medicine, Taipei Veterans General Hospital, Taipei, 11217 Taiwan; 2 Department of Pathology, Taipei Veterans General Hospital, Taipei, 11217 Taiwan; 3 School of Medicine, National Yang-Ming University, Taipei, 11221 Taiwan; 4 Jen Mei Clinic, New Taipei City, 24759 Taiwan

## Abstract

BK virus nephropathy (BKVN) and allograft rejection are two distinct disease entities which occur at opposite ends of the immune spectrum. However, they coexist in renal transplant recipients. Predisposing factors for this coexistence remain elusive. We identified nine biopsy-proven BKVN patients with coexisting acute rejection, and 21 patients with BKVN alone. We retrospectively analyzed the dosage and blood concentrations of immunosuppressants during the 3-month period prior to the renal biopsy between the two patient groups. Compared to the BKVN alone group, renal function was noticeably worse in the coexistence group (*p* = 0.030). Regarding the dose and average drug level of immunosuppressants, there was no difference between the two groups. Interestingly, the coefficient of variance of tacrolimus trough blood level was noticeably higher during the 3-month period prior to the renal biopsy in the coexistence group (*p* = 0.010). Our novel findings suggest that a higher variability of tacrolimus trough level may be associated with the coexistence of BKVN and acute rejection. Since the prognosis is poor and the treatment is challenging in patients with coexisting BKVN and acute rejection, transplant clinicians should strive to avoid fluctuations in immunosuppressant drug levels in patients with either one of these two disease entities.

## Introduction

Although BK virus nephropathy (BKVN; B.K. w﻿as ﻿originated from a patient's initials in ﻿1971^1^) and allograft rejection are two distinct disease entities which occur at opposite ends of the immune spectrum, they coexist in renal transplant recipients. BKVN usually results from excessive immunosuppression therapy^2, 3^ and predisposes to graft dysfunction or graft loss^4, 5^. Reducing the dosage of immunosuppressants is the mainstay of management^6, 7^. Over-suppression of the immune system promotes BK viral growth, while insufficient immunosuppression results in acute rejection.

BKVN is an important cause of graft failure, and may affect up to 15% of renal transplant recipients. Diagnosing BKVN is difficult, especially when protocol biopsies are lacking, and it is even more challenging if BKVN coexists with acute rejection. Careful pathological interpretation and differential diagnoses are essential for an accurate diagnosis.

Both BKVN and acute rejection present with a decline in renal function^8, 9^, however very different management strategies are needed, and a renal biopsy may be required for a definite diagnosis. BKVN and acute rejection may occur at the same time, with a reported incidence ranging from 1%~24%^10–12^. The mechanism remains elusive, and only case reports or descriptive studies have been published to date^10–20^. A recent study reported an unfavorable clinical outcome when BKVN and acute rejection coexist^15^. Nevertheless, the pathogenesis and predisposing factors for the coexistence of BKVN and acute rejection remain unknown.

It has been reported that fluctuations in the blood levels of tacrolimus are strongly related to poor kidney graft function^21–25^. In addition, high drug level variability has also been reported to promote donor-specific antibody development and increased graft rejection rates^26–28^. We hypothesized that fluctuations in immunosuppressant drug level may be associated with the coexistence of BKVN and acute rejection. We aimed to identify the predisposing factors in order to help transplant clinicians prevent the development of this disease and eventually improve allograft outcomes.

## Results

### Baseline characteristics of the study subjects

Thirty patients with biopsy-proven BKVN were enrolled, including nine in the coexistence group and 21 in the BKVN alone group. Table 1 summarizes the demographic characteristics. The mean age was 48.2 ± 9.2 years, and 66.7% of the patients were male. All grafts were from deceased donors, and renal biopsies were performed 1.8 ± 1.3 years post-transplantation. Among the patients, 56.7% had hypertension, and 20% had diabetes mellitus. The average serum creatinine level of all patients at the time of biopsy was 2.5 ± 1.1 mg/dL. The eGFR calculated using the CKD-EPI and simplified MDRD formulae were both noticeably lower in the coexistence group (eGFR-C, *p* = 0.029, and eGFR-M, *p* = 0.030). Otherwise, there were no differences in the listed characteristics. In addition, two patients in the coexisting group were diagnosed with acute cellular rejection, one with acute antibody-mediated rejection, and the other with combined cellular and humoral rejection. There was no difference in the type of acute rejection.

**Table 1:** Demographic characteristics between coexisting BKVN and AR (*n* = 9) and BKVN alone (*n* = 21) groups.

Factor	All	Coexisting BKVN and AR (*n* = 9)	BKVN alone (*n* = 21)	*p* Value
Patient number (*n*)	30	9	21	
Age (year)	48.2 ± 9.2	50.9 ± 9.1	47.1 ± 9.2	0.301
Gender (male %)	66.7	55.6	71.4	0.431
Deceased donor (%)	100.0	100.0	100.0	1.000
Timing of renal biopsy after transplantation (year)	1.8 ± 1.3	1.7 ± 0.8	1.9 ± 1.5	0.717
PRA, class I [median (IQR)]	0.0 (0.0–0.0)	0.0 (0.0–15.8)	0.0 (0.0–0.0)	0.842
PRA, class II [median (IQR)]	0.0 (0.0–0.0)	0.0 (0.0–0.0)	0.0 (0.0–0.0)	1.000
Comorbidities				
Diabetes mellitus (%)	20.0	22.2	19.0	1.000
Hypertension (%)	56.7	66.7	52.4	0.691
Congestive heart failure (%)	10.0	11.1	9.5	1.000
Laboratory data at biopsy				
Albumin (g/dL)	4.1 ± 0.5	3.8 ± 0.5	4.2 ± 0.5	0.102
Creatinine (mg/dL)	2.5 ± 1.1	3.1 ± 1.5	2.2 ± 0.8	0.121
eGFR-C (mg/dL)	32.6 ± 12.1	25.5 ± 13.0	35.7 ± 10.5	0.029*
eGFR-M (mg/dL)	32.4 ± 11.5	25.5 ± 12.4	35.4 ± 10.0	0.030*
Hb (g/dL)	10.7 ± 1.9	10.0 ± 2.0	11.0 ± 1.8	0.166
ALC (per cumm)	921.6 ± 530.4	838.9 ± 437.4	958.9 ± 573.9	0.582
Patient Outcomes				
Follow-up period (years post renal biopsy)	5.7 ± 2.5	6.2 ± 2.4	5.4 ± 2.5	0.443
Graft failure requiring RRT (*n*; %)	12; 40.0	4; 44.4	8; 38.1	1.000
Time of graft failure (years post renal biopsy)	5.8 ± 1.8	6.5 ± 2.6	5.6 ± 1.3	0.536
All-cause mortality (*n*; %)	5; 16.7	3; 33.3	2; 9.5	0.143
Time of mortality (years post renal biopsy)	5.9 ± 2.7	7.5 ± 2.2	3.5 ± 1.0	0.100

Values are expressed as mean ± SD, median (IQR), or percentage as appropriate. Abbreviations: BKVN, BK virus nephropathy; AR, acute rejection; PRA, panel reactive antibody; eGFR-C, estimated glomerular filtration rate by CKD-EPI formula; eGFR-M, estimated glomerular filtration rate by simplified MDRD formula; Hb, hemoglobin; ALC, absolute lymphocyte count; RRT, renal replacement therapy. *p < 0.05.

### Immunosuppressive agent regimens

We reviewed all immunosuppressant regimens during the 3-month period prior to the renal biopsies (Table 2). Corticosteroids, tacrolimus, mycophenolate mofetil, or sirolimus were prescribed in all 30 patients, and none received cyclosporine. The daily dose, trough blood level, and body weight of each patient were recorded. There was no difference between the two patient groups in daily dose per body weight or mean drug level of tacrolimus or sirolimus.

**Table 2:** Regimens and blood levels of immunosuppressive agents.

Factor	All	Coexisting BKVN and AR (*n* = 9)	BKVN alone (*n* = 21)	*p* Value
Patient number (*n*)	30	9	21	
Time post transplantation (year)	1.8 ± 1.3	1.6 ± 0.8	1.8 ± 1.5	0.728
IS regimens within 3 months prior to renal biopsy				
Corticosteroids, oral (*n*; %)	27; 90.0	8; 88.9	19; 90.5	1.000
Daily dose/BW (mg/kg/day)	0.125 ± 0.133	0.175 ± 0.219	0.104 ± 0.069	0.366
Mycophenolate mofetil (*n*; %)	27; 90.0	8; 88.9	19; 90.5	1.000
Daily dose/BW (mg/kg/day)	16.731 ± 7.652	20.193 ± 8.756	18.119 ± 9.927	0.138
Tacrolimus (*n*; %)	28; 93.3	8; 88.9	20; 95.2	0.517
Daily dose/BW (mg/kg/day)	0.063 ± 0.044	0.058 ± 0.033	0.065 ± 0.048	0.701
Sirolimus (*n*; %)	11; 36.7	7; 77.8	4; 19.0	0.004*
Daily dose/BW (mg/kg/day)	0.013 ± 0.019	0.027 ± 0.021	0.007 ± 0.015	0.023*
**Combined use of tacrolimus and sirolimus (** ***n*** **; %)**	**7; 23.3**	**4; 44.4**	**3; 14.3**	**0.153**
**Switch between tacrolimus and sirolimus (** ***n*** **; %)**	**4; 13.3**	**2; 22.2**	**2; 9.5**	**0.563**
**Switch from tacrolimus to sirolimus (** ***n*** **; %)**	**3; 10.0**	**2; 22.2**	**1; 4.8**	**0.207**
**Switch from sirolimus to tacrolimus (** ***n*** **; %)**	**1; 3.3**	**0; 0.0**	**1; 4.8**	**1.000**
**Tacrolimus serum trough level, all (** ***n*** **)**	**27**	**7**	**20**	
**Month, out-of-range, average (** ***n*** **)**	**2.07**	**1.86**	**2.15**	**0.746**
**Month, total, average (** ***n*** **)**	**7.15**	**5.57**	**7.70**	**0.237**
**Out-of-range, average (%)**	**26.3**	**36.5**	**22.8**	**0.133**
Tacrolimus serum trough level within 3 months prior to renal biopsy (*n*)	25	6	19	
Mean (ng/mL)	5.46	4.33	5.82	0.236
Standard deviation (ng/mL)	1.44	1.82	1.33	0.320
Coefficient of variance (%)	28.6	43.3	24.0	0.010*
**Tacrolimus serum trough level within 6 months prior to renal biopsy (** ***n*** **)**	**21**	**4**	**17**	
**Mean (ng/mL)**	**5.73**	**5.45**	**5.80**	**0.771**
**Standard deviation (ng/mL)**	**1.90**	**1.86**	**1.92**	**0.927**
**Coefficient of variance (%)**	**33.4**	**33.8**	**33.4**	**0.960**
**Tacrolimus serum trough level within 9 months prior to renal biopsy (** ***n*** **)**	**17**	**4**	**13**	
**Mean (ng/mL)**	**5.47**	**5.22**	**5.54**	**0.707**
**Standard deviation (ng/mL)**	**2.17**	**1.96**	**2.23**	**0.641**
**Coefficient of variance (%)**	**39.6**	**38.7**	**39.9**	**0.883**
**Tacrolimus serum trough level within 12 months prior to renal biopsy (** ***n*** **)**	**7**	**1**	**6**	
**Mean (ng/mL)**	**5.42**	**3.09**	**5.81**	**0.213**
**Standard deviation (ng/mL)**	**2.40**	**1.63**	**2.53**	**0.439**
**Coefficient of variance (%)**	**45.8**	**52.9**	**44.6**	**0.618**
**Tacrolimus serum trough level within 15 months prior to renal biopsy (** ***n*** **)**	**5**	**1**	**4**	
**Mean (ng/mL)**	**5.27**	**3.38**	**5.74**	**0.390**
**Standard deviation (ng/mL)**	**2.23**	**1.89**	**2.32**	**0.696**
**Coefficient of variance (%)**	**44.7**	**55.8**	**41.9**	**0.454**
Sirolimus serum trough level within 3 months prior to renal biopsy (*n*)	7	4	3	
Mean (ng/mL)	7.05	7.53	6.41	0.605
Standard deviation (ng/mL)	2.96	4.06	1.50	0.244
Coefficient of variance (%)	40.7	56.8	19.3	0.238
**Sirolimus serum trough level within 6 months prior to renal biopsy (** ***n*** **)**	**4**	**1**	**3**	
**Mean (ng/mL)**	**7.07**	**10.37**	**5.97**	**0.342**
**Standard deviation (ng/mL)**	**1.80**	**1.89**	**1.77**	**0.951**
**Coefficient of variance (%)**	**27.8**	**18.2**	**31.0**	**0.562**
**Sirolimus serum trough level within 9 months prior to renal biopsy (** ***n*** **)**	**3**	**1**	**2**	
**Mean (ng/mL)**	**8.09**	**9.88**	**7.20**	**0.173**
**Standard deviation (ng/mL)**	**1.92**	**1.84**	**1.97**	**0.794**
**Coefficient of variance (%)**	**24.3**	**18.6**	**27.2**	**0.190**
**Sirolimus serum trough level within 12 months prior to renal biopsy (** ***n*** **)**	**2**	**0**	**2**	
**Mean (ng/mL)**	**8.06**		**8.06**	
**Standard deviation (ng/mL)**	**2.12**		**2.12**	
**Coefficient of variance (%)**	**26.4**		**26.4**	
**Sirolimus serum trough level within 15 months prior to renal biopsy (** ***n*** **)**	**2**	**0**	**2**	
**Mean (ng/mL)**	**8.37**		**8.37**	
**Standard deviation (ng/mL)**	**2.10**		**2.10**	
**Coefficient of variance (%)**	**25.2**		**25.2**	

Values are expressed as mean ± SD or percentage. Abbreviations: BKVN, BK virus nephropathy; AR, acute rejection; BW, body weight; IS, immunosuppressants. *p < 0.05.

As shown in Table 2, the combined use of tacrolimus and sirolimus in the two patient groups was 44.4% and 14.3%, respectively, with no statistical difference (*p* = 0.153). Two patients switched from tacrolimus to sirolimus in the coexistence group, and one switched in the BKVN alone group (22.2% *vs*. 4.8%, *p = *0.207). Only one of the 30 patients switched from sirolimus to tacrolimus (in the BKVN alone group).

### Blood levels of immunosuppressive agents

We analyzed the mean dosage and coefficient of variance (CV) of trough blood levels of immunosuppressants during different vintages prior to the renal biopsy (3, 6, 9, 12, and 15 months) in the two groups. The tacrolimus trough blood level during the 3-month period prior to the renal biopsy had a noticeable larger fluctuation (*p* = 0.010). The CVs of sirolimus trough blood levels during the 3-month period in the two groups were 56.8% and 19.3%, which showed a higher variability in the coexistence group but did not achieve statistical significance (*p* = 0.238). There were no noticeable differences between the two groups in the variability of tacrolimus/sirolimus levels of the other vintages (6, 9, 12, and 15 months).

With regards to the tacrolimus serum trough level, the percentage of out-of-range values was higher in the coexistence group than in the BKVN group (36.5% *vs*. 22.8%, *p = *0.133), although with no statistical significance. Table 3 shows the results of the adjusted logistic regression analysis. Compared to the BKVN alone group, the CV of tacrolimus trough blood level was noticeably higher in the coexistence group (odds ratio 1.068, *p* = 0.039).

**Table 3:** Determinants of coexisting BK virus nephropathy and acute rejection.

Factor	Crude OR	95% CI		*p* Value	Adjusted OR^#^	95% CI		*p* Value
		Lower	Upper			Lower	Upper	
Tacrolimus use (reference: no)	0.400	0.022	7.201	0.534	**0.365**	**0.017**	**8.021**	**0.522**
Tacrolimus serum trough level within 3 months prior to renal biopsy								
Mean (ng/mL)	0.770	0.499	1.188	0.237	**0.839**	**0.544**	**1.294**	**0.428**
Standard deviation (ng/mL)	1.521	0.663	3.489	0.322	**1.825**	**0.711**	**4.680**	**0.211**
Coefficient of variance (%)	1.079	1.007	1.156	0.031*	**1.068**	**1.003**	**1.137**	0.039*
Sirolimus use (reference: no)	14.875	2.198	100.656	0.006*	**10.490**	**1.429**	**76.975**	0.021*
Sirolimus serum trough level within 3 months prior to renal biopsy								
Mean (ng/mL)	1.239	0.631	2.429	0.534	**1.377**	**0.637**	**2.979**	**0.416**
Standard deviation (ng/mL)	1.741	0.691	4.387	0.239	**1.706**	**0.568**	**5.120**	**0.341**
Coefficient of variance (%)	1.065	0.949	1.194	0.283	**1.067**	**0.929**	**1.226**	**0.356**

^#^Adjusted models were adjusted for a propensity score consisting of recipient age, gender, deceased donor, transplantation vintage, diabetes mellitus, hypertension, congestive heart failure, albumin, estimated glomerular filtration rate according to the CKD-EPI formula, and absolute lymphocyte count. Abbreviations: OR, odds ratio; CI, confidence interval. *p < 0.05.

### Patient outcomes

The average post-biopsy follow-up duration was 5.7 ± 2.5 years. The grafts failed in 40% of the patients, and renal replacement therapy was initiated 5.8 ± 1.8 years after the renal biopsy among these patients. Overall, 16.7% of the patients died 5.9 ± 2.7 years after the renal biopsy. As shown in Table 1, the graft failure (44.4% *vs*. 38.1%) and all-cause mortality (33.3% *vs*. 9.5%) rates tended to be higher in the coexistence group than in the BKVN alone group. However, the differences did not reach statistical significance, probably due to the small scale of the present cohort.

## Discussion

This study identified that the coexistence of BKVN and acute rejection was noticeably associated with a fluctuating tacrolimus trough blood level during the 3-month period prior to the renal biopsy but not with the dose or the average drug level of any immunosuppressant. Although the mean tacrolimus serum trough level during the 3 months prior to the renal biopsy was 1.5 ng/mL higher in the BKVN alone group (Table 2), the CV of tacrolimus showed a more noticeable difference (*p = *0.010). In addition, sirolimus level variability also seemed to be higher in the coexistence group but without statistical significance, probably because fewer patients used sirolimus in our cohort. Small-scale studies have described the coexistence of BKVN and acute rejection, most of which have focused on the clinical course^10–15, 17, 18, 20^ with several emphasizing the therapy^16, 19^. In addition, pathologists have made efforts to accurately diagnose the coexistence of BKVN and acute rejection using strategies that may be completely opposite^10, 20, 29^. However, the mechanism is still unknown, and our study is the first to identify its determinants.

BKVN is an important cause of graft failure, with a reported incidence of up to 15%^30^. BK virus originates either from the donor or recipient, and is potentiated by augmented immunosuppression^31^. As a result, the viral load and allograft inflammation are attenuated once the dose of immunosuppressants is reduced^32, 33^. Other risk factors such as male gender, older recipient age, prolonged cold ischemic time, ureteral stent placement, rejection episodes, human leukocyte antigen (HLA) mismatch, and induction immunosuppressive therapy have been reported, suggesting that the pathogenesis of BKVN is multifactorial^31^. The virus can be detected using urine cytology or blood real time polymerase chain reaction analysis, however a renal biopsy remains the most reliable method. Lowering the dosage of immunosuppressants is the principle management strategy, and the use of anti-viral therapy such as cidofovir or leflunomide is no longer routinely recommended^30^.

The reported incidence of coexisting BKVN and acute rejection ranges from 1% to 24%^10–12^. Both BKVN and acute rejection present with a decline in renal function^8, 9^, however their management strategies are quite different, and a renal biopsy may be required for a definite diagnosis. Due to the focal nature of BKVN, two allograft biopsy cores are recommended for better sensitivity^34^. The pathological examinations should include two parts to diagnose BKVN: viral cytopathic changes, and grading of interstitial fibrosis/tubular atrophy/fibrosis according to the Banff scheme^35^. The diagnosis is challenging if BKVN occurs with Banff type 1 rejection. Inflammatory cell infiltration may represent immune reactions to virus nephritis, which then leads to difficulty in distinguishing BKVN from tubulointerstitial rejection^16^. Immunohistochemistry and electron microscopy may help in the diagnosis of BKVN, whereas specific pathological findings such as endarteritis, fibrinoid vascular necrosis, glomerulitis, HLA-DR tubular expression and C4d deposits along peritubular capillaries may help in the diagnosis of acute rejection^31^. Despite the retrospective nature of this study, mandatory electron microscope examinations were conducted for all renal biopsy specimens at our institute, and all pathological findings were reviewed and validated by a second senior pathologist to confirm the diagnosis in our cohort.

Immunosuppressant blood level monitoring is crucial in transplant recipient care because of the concentration-effect relationship, the narrow therapeutic window, and the nephrotoxicity of calcineurin inhibitors^36^. Fluctuations in tacrolimus level may be caused by many pharmacokinetic and pharmacogenetic factors. From the patient perspective, ingested food, daily drug-drug interactions and adherence to immunosuppressive drugs are important issues^37^. Grapefruit may also increase the exposure to tacrolimus by inhibiting the hepatic activity of cytochrome P450 3A4^38^. Macrolide antibiotics, calcium channel blockers, anti-epileptic drugs and anti-fungal azoles are common medications^39^, and over-the-counter drugs and herbal medicine may also explain the fluctuations. In addition, genetic differences have been reported to play a role in intra-patient variability^40, 41^. Suboptimal compliance to immunosuppressive drug regimens is known to result in poor long-term renal outcomes^42^. From the clinical perspective, different analytical methods for tacrolimus and generic tacrolimus substitution may influence the drug level^37^. In 2015, Shuker *et al*. analyzed tacrolimus intra-patient variability (IPV) in 167 patients using tacrolimus once- or twice-daily^43^. Their data showed a wide range of IPV with some individuals having a tacrolimus IPV of 5%, and others having a variability of 50%. On average, tacrolimus IPV was between 15% and 30%. In our study, the CV of tacrolimus serum trough levels during the 3-month period prior to the renal biopsy was 28.6%, which is compatible with Shuker’s data.

It has been reported that higher intra-individual variability of tacrolimus is strongly correlated with poor kidney graft function and higher chronic rejection rates^21–25^. The relationship between long-term transplant renal outcomes and intra-patient variability of tacrolimus level was first reported by Borra *et al*.^21^, who found that a higher variability in intra-patient tacrolimus level was related to 1-year post-transplant graft function decline. A study with a larger sample size demonstrated that a greater variation in intra-patient tacrolimus level was associated with late allograft rejection 1 year after transplantation, and also that a larger standard deviation in tacrolimus level was associated with inferior graft outcomes^23^. Taken together, these findings suggest that high variability in tacrolimus level is related to worse graft outcomes.

A higher acute rejection rate has also been reported in patients with a greater variation in intra-patient tacrolimus level^27^. In addition, a biopsy-proven pediatric study also demonstrated that a high CV of tacrolimus level was noticeably correlated with a high risk of allograft rejection^26^. Another study reported that fluctuations in drug level promoted the development of donor-specific antibodies and that this was a strong risk factor for increased death-censored graft loss. The authors postulated that high drug level variability represented low exposure to immunosuppressants, even if the mean drug level remained within the target range. They concluded that alloimmune responses triggered by insufficient immunosuppression may lead to the development of donor-specific antibodies^28^. This hypothesis reflects our findings, indicating that patients with high drug level variability are also at risk of excessive immunosuppression, which then predisposes them to BK virus infection.

**Figure 1:**
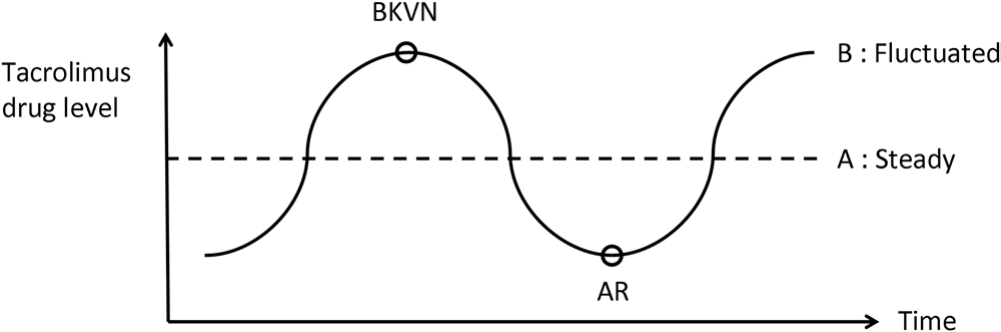
Proposed model for the pathogenesis of coexisting BKVN and AR. Abbreviations: BKVN, BK virus nephropathy; AR, acute rejection.

The mechanism of the coexistence of BKVN and acute rejection is still not completely understood. It is possible that over-exposure to tacrolimus may induce BK virus infection, while insufficient tacrolimus treatment may result in acute rejection in the same allograft (Fig. 1, line B). Our results also indicated a potential association between fluctuations in sirolimus level and the coexistence of BKVN and acute rejection. The CV of sirolimus level was 56.8% in the coexistence group and 19.3% in the BKVN alone group in this study. Although the difference was not statistically significant, probably due to a relatively low patient number, fluctuations in sirolimus level might also be correlated with the coexistence of BKVN and acute rejection. A recent study concluded that BK virus reactivation is associated with immune responses to kidney-specific self-antigens including fibronectin and collagen type IV, and that such immune responses may subsequently increase the risk of acute rejection through unclear mechanisms^44^. This study echoes our findings, and further studies are warranted to elucidate this issue.

There are several limitations to the present study. First, the sample size was small due to the rareness of this disease. However, this is the first study to identify that high drug level variability is a predisposing factor. Second, due to the retrospective design of this study, no protocol biopsies were available and unified immunosuppressive regimens were not used for all of the patient. However, the diagnoses were all based on kidney pathology instead of merely via urine or blood examinations.

In conclusion, our results clearly demonstrated that fluctuations in tacrolimus level were noticeably associated with the coexistence of BKVN and acute rejection. In addition, the CV of sirolimus trough level also seemed to be higher in the coexistence group. Since coexisting BKVN and acute rejection may lead to poor clinical outcomes, transplant clinicians need to manage the immunosuppressant dosage prudently in patients with either one of these two disease entities, particularly when protocol biopsies are lacking.

## Methods

### Study protocol and subjects

**Figure 2:**
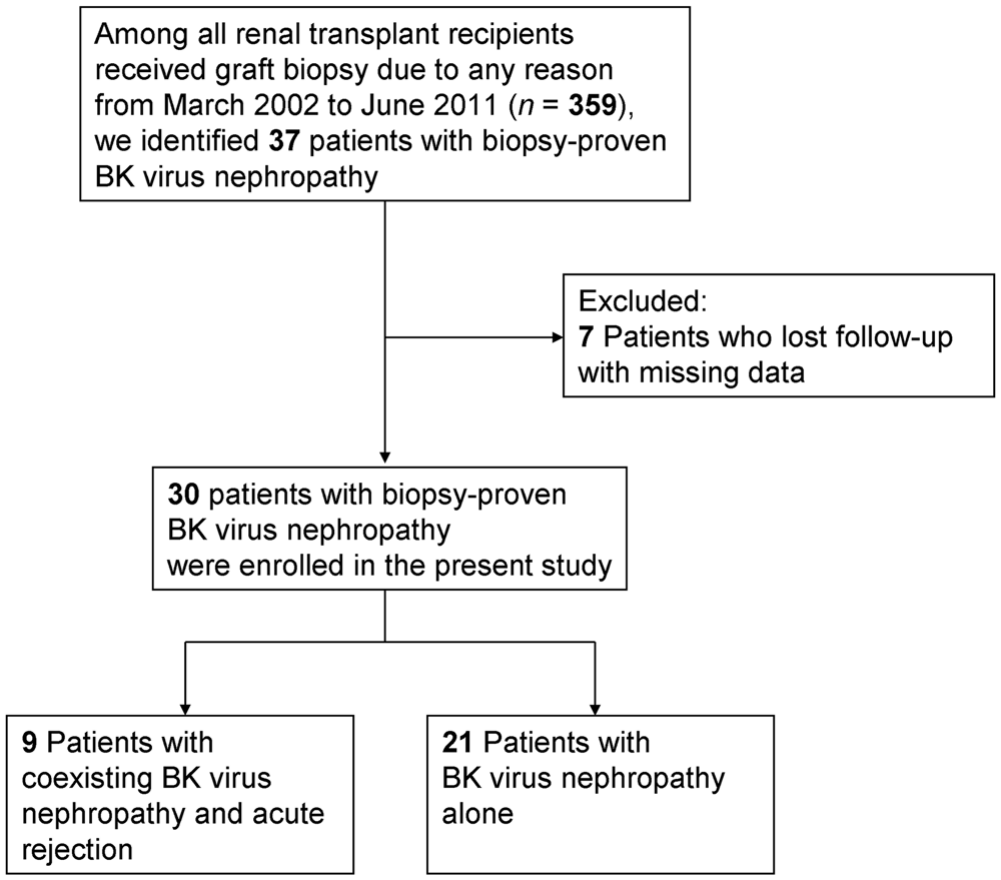
Flow diagram of study enrollment.

This is a retrospective observational study on renal allograft recipients in a single institute. We reviewed the records of all patients who underwent renal transplantation at Taipei Veteran General Hospital, a tertiary-care referral center in Taiwan, between March 2002 and June 2011. During this period, we performed 359 percutaneous graft kidney needle biopsies, and 37 patients were diagnosed with BKVN. Patients who were lost to follow-up and had missing data (*n* = 7) were excluded. The remaining 30 patients were enrolled in this study and categorized into two groups: those with coexisting BKVN and acute rejection (*n* = 9), and those with BKVN alone (*n* = 21) (Fig. 2).

We collected demographic information from medical records, including age, gender, comorbidities, donor factors, panel reactive antibodies of the recipient, cause of end-stage renal disease of the native kidney, duration of dialysis before transplant, timing of transplant and biopsy, and pathological diagnosis of the graft kidney. Immediately before the biopsy, we recorded laboratory data including serum creatinine, estimated glomerular filtration rate (eGFR-C using the CKD-EPI formula^45^, and eGFR-M using the simplified MDRD formula^46^), and levels of hemoglobin and albumin. Patient outcomes included graft failure requiring renal replacement therapy and all-cause mortality.

All protocols were approved by the Institutional Review Board of the institute before the study began, and the protocols conformed to the ethical guidelines of the *Helsinki* Declaration. The need for informed consent was waived because of the retrospective nature of the study.

### The immunosuppressant regimens

After renal transplantation, the recipients were regularly followed up at our institute on at least a monthly basis. The immunosuppressive protocol and the strategy of dose reduction at our hospital were determined by each attending physician. In general, sequential triple therapy consisting of glucocorticoids, calcineurin inhibitors such as tacrolimus or cyclosporine, mycophenolate mofetil, or sirolimus in various combinations was used for maintenance therapy. We recorded the regimen, daily and accumulative dosage of immunosuppressants as well as the respective drug trough blood level. Daily dose per body weight was calculated as total accumulative dose divided by the total treatment duration and body weight.

The combined use of immunosuppressants was defined as an immunosuppressive regimen consisting initially of tacrolimus and then the addition of sirolimus for more than 1 month, or vice versa. The switch of immunosuppressants was defined as an immunosuppressive regimen initially consisting of tacrolimus and then switching to sirolimus, or vice versa. An evidence-based serum tacrolimus trough level target is used at our institute according to the post-transplant period as follows^47–52^: 6–15 ng/mL within 3 months after kidney transplantation; 4–12 ng/mL during 3–12 months post-transplant; and 3–7 ng/mL after 12 months post-transplant. The sirolimus trough level targets were set as 8–12 ng/mL within 3 months post-transplant, and 5–10 ng/mL after 3 months post-transplant.

### Pathological findings of the graft biopsy

The renal pathological report of each patient was examined and diagnosed by two senior pathologists specializing in kidney transplant pathology. The histological features of BKVN were examined including cytopathic changes, interstitial inflammation/tubular atrophy and ancillary tests such as SV40 immunohistochemistry, immunofluorescence or electron microscopy. The diagnosis of rejection was made according to the Banff ’07 classification of renal allograft biopsy^31, 35, 53^.

### Statistical analysis

The chi square test was used for comparisons of categorical variables. Continuous variables were described as mean ± standard deviation for normally distributed data, and as median (interquartile range [IQR]) for non-normally distributed data. Continuous variables were analyzed using the Student’s *t* test or Mann-Whitney *U* test as appropriate. We compared the dosage of tacrolimus/sirolimus and the trough blood level at 3, 6, 9, 12, and 15 months prior to the renal biopsy between the two patient groups. We used coefficient of variance (CV) to quantify fluctuations in drug blood level. CV was defined as the ratio of the standard deviation (SD) to the mean, and expressed as a percentage using the formula: CV% = (SD/Mean) × 100%. The serum level of the immunosuppressant was regarded as being “out-of-range” if it was not within the aforementioned target level of our institute^47–52^. The percentage of out-of-range values was calculated as the number of months of being out-of-range divided by the total number of months measured. To identify the determinants of coexisting BKVN and acute rejection, we using logistic regression analysis. In view of the small size of the present cohort, a propensity score was generated and included in the multivariate logistic regression analysis. The propensity score was calculated using a logistic model consisting of possible confounding variables including recipient age, gender, deceased donor, transplantation vintage, diabetes mellitus, hypertension, congestive heart failure, albumin, eGFR-C, and absolute lymphocyte count. All data were analyzed using Statistical Package for the Social Sciences (SPSS) version 18.0 (SPSS, Chicago, USA). All probabilities were two-tailed, and a *p* value of less than 0.05 was considered to be statistically significant.
